# Correlates of objectively measured overweight/obesity and physical activity in Kenyan school children: results from ISCOLE-Kenya

**DOI:** 10.1186/1471-2458-14-436

**Published:** 2014-05-09

**Authors:** Stella K Muthuri, Lucy-Joy M Wachira, Vincent O Onywera, Mark S Tremblay

**Affiliations:** 1 Children’s Hospital of Eastern Ontario Research Institute, 401 Smyth Road, Ottawa, Ontario K1H 8L1, Canada; 2 University of Ottawa, Ottawa, Ontario, Canada; 3 Kenyatta University, Nairobi, Kenya

**Keywords:** Overweight, Obesity, Physical activity, School children, Kenya

## Abstract

**Background:**

Childhood overweight/obesity and inadequate physical activity burden Western countries, and now, pose a growing threat to the health of children in low and middle income countries. Behavioural transitions toward more sedentary lifestyles coupled with increased consumption of high calorie foods has resulted in rising proportions of overweight/obesity and decreasing levels of physical activity in school-aged children. The objective of this study was to determine the prevalence and to investigate factors associated with overweight/obesity and physical activity in Kenyan children aged 9 to 11 years.

**Methods:**

Body composition and physical activity measures of participating children were accomplished by anthropometric assessment, accelerometry, and administration of questionnaires related to diet and lifestyle, and the school and neighbourhood environments. Data collection was conducted in the city of Nairobi as part of a larger International Study of Childhood Obesity, Lifestyle and Environment.

**Results:**

A total of 563 participants (46.5% boys, 53.5% girls) were included in the analyses. Of these, 3.7% were underweight, 14.4% were overweight, and 6.4% were obese based on WHO cut-points. Mean daily sedentary time was 398 minutes, time spent in light physical activity was 463 minutes, and time spent in moderate-to-vigorous physical activity was 36 minutes based on activity cut-points developed by Treuth *et al*. Only 12.6% of participating children were meeting the recommendation of ≥ 60 minutes of daily moderate-to-vigorous physical activity, and 45.7% of participants used active transportation to/from school. Increasing parental education level, total annual household income, and attending a private rather than public school were associated positively with being overweight/obese and negatively with meeting physical activity guidelines.

**Conclusions:**

This study provided the evidence for an existing prevalence of childhood overweight/obesity in Nairobi. Children were spending a considerable amount of time in sedentary and light intensity physical activity, with few meeting physical activity guidelines. Higher socioeconomic status and parental education attainment were associated with a higher likelihood of children being overweight/obese and a lower likelihood of children meeting the physical activity recommendations. Interventions and strategies should be attentive to the potential health consequences of lifestyle transitions resulting from urbanisation and economic prosperity.

## Background

Physical activity and nutritional transitions around the world have resulted in a shift towards more sedentary lifestyles, increased consumption of high calorie foods, and rising proportions of overweight and obesity (overweight/obesity). These transitions have led to increases in the occurrence of modifiable non-communicable diseases such as diabetes, hypertension, heart disease, and some forms of cancer [1]. Physical inactivity and overweight/obesity are classified as the fourth and fifth leading causes of global mortality, and two of the greatest health challenges and determinants for various chronic diseases [1-4]. Of major concern is the potential for lifelong health consequences in children and youth, who have not been spared from the effects of these behavioural transitions. Childhood overweight/obesity is significantly associated with increased risk of obesity, physical morbidity, and premature mortality in adulthood [5-8]. However, children who attain a healthy weight by adolescence have better cardiovascular disease risk factor profiles compared to those remaining overweight [6].

Global physical activity guidelines recommend that children and youth 5–17 years of age should accumulate an average of at least 60 minutes of daily moderate-to-vigorous physical activity (MVPA) in order to improve or maintain a healthy cardiorespiratory, fitness, and body composition profile [9].

Unfortunately, childhood overweight/obesity and inadequate physical activity remain a challenge in high income countries, and now pose a growing public health threat to children in low and middle income countries, particularly in urbanised areas [10,11]. While the health benefits of maintaining healthy active lifestyles are well established, in many Sub-Saharan African countries including Kenya, declines in habitual physical activity (e.g. traditional practices of walking long distances, manual labour) and subsequent increases in sedentary behaviour (e.g. motorised transport, desk jobs) have been observed [10-13]. Whereas physical activity and nutritional transitions have been well documented in high income countries, in Kenya, there is a lack of nationally representative data on the prevalence of childhood overweight/obesity and levels of physical activity among school-aged children. Even fewer studies have examined correlates or other factors associated with childhood overweight/obesity and physical activity. However, a growing number of research studies that are not nationally representative have shown emerging evidence of an overweight/obesity and inadequate physical activity problem in school-aged children in Kenya, predominantly in urban living children [14-20].

The primary objective of this study was to determine the prevalence of and investigate factors associated with overweight/obesity and physical activity in children aged 9 to 11 years, recruited from public and private schools in Nairobi. Data analysis sought to investigate the relationships between overweight/obesity or physical activity and factors at the individual, parental, household, neighbourhood, and school environment levels. Examination of these factors was guided by the socioecological model for creating active living communities proposed by Sallis *et al*., which identifies potential environmental and policy influences on four broad domains of active living: household activities (e.g. household environment and facilities), active recreation (e.g. neighbourhood environment and facilities), active transport (e.g. neighbourhood walkability and safety), and occupational activities (e.g. the schools environment and policies) [21]. It was hypothesised that the results of this study would show further evidence of an emerging overweight/obesity and inadequate physical activity threat in school-aged children in Kenya, while also providing novel evidence of correlates of these outcomes in this African context.

## Methods

Body composition and physical activity measures of the participating children were completed by direct anthropometric assessment and questionnaires related to diet and lifestyle, and the school and neighbourhood environments. Data collection was conducted as part of the International Study of Childhood Obesity, Lifestyle and Environment (ISCOLE) [22].

### The ISCOLE project

The primary aim of ISCOLE was to investigate the influence of behavioural settings and the physical, social, and policy environments on the observed relationship between lifestyle characteristics and weight status in approximately 500 children from each of the included 12 countries (representing the major regions of the world) [22]. Given that much of the knowledge available on the influence of lifestyle behaviours on overweight/obesity and physical activity is informed by studies conducted in North America and Europe [23,24], there is a pressing need to determine the robustness of these relationships across all global regions [22]. Details of the ISCOLE study protocol are provided elsewhere [22]. Data collection was conducted in the urban city of Nairobi, for ISCOLE-Kenya’s assessments following ethical approval from Kenyatta University, the Nairobi City Council, and the National Council for Science and Technology.

### Study design and population

**Figure 1:**
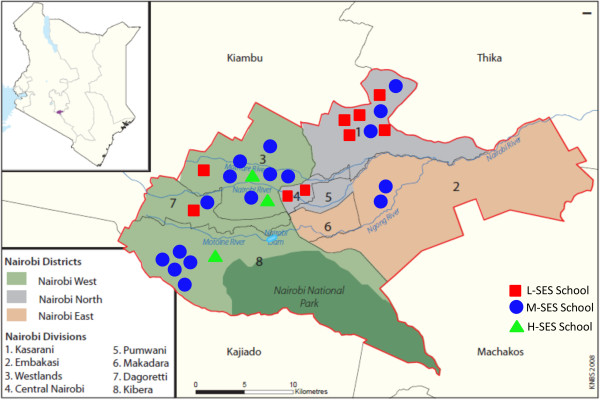
Geographical representation of participating schools by Divisions of Nairobi.

Recruitment targeted a sex-balanced sample of 500 children between the ages of 9 and 11 years. The primary sampling frame was a convenience sample of non-boarding public and private schools in Nairobi as shown in Figure 1. Schools were recruited proportional to the distribution of public and private school attendance. Non-compliant schools were replaced with the next randomly selected school. Type of school, public verses private, was an indicator of lower and higher socioeconomic status (SES) respectively. The secondary sampling frame was classrooms in the recruited schools that best yielded a final sample with minimal variability around 10 years of age. Children included in this study were typically in classes 4 or 5. Depending on the size of the classroom, 30 to 50 children were approached to participate in the study and were sent home with an introduction letter and consent form for their parents or guardians to review and give consent. Children were thereafter asked to provide assent to participate in the study. As per the study protocol, data collection was conducted for a full school year (2012, excluding the month long school holiday breaks in April, August, and December) in order to allow for examination of any seasonal differences [22].

### Direct measures (anthropometry)

Extensively trained research staff measured standing and sitting height of participating children using the Seca 213 portable stadiometer (Birmingham, United Kingdom), with the participant as erect as possible and head positioned in the Frankfort horizontal plane [22]. The participant’s weight, body fat percentage, and bioelectrical impedance were measured using a portable Tanita Body Composition Analyser (SC-240, Illinois, USA), after all outer clothing, heavy pocket items, shoes, and socks were removed [22]. Waist circumference measurements were made on exposed skin at the end of a normal expiration using a non-elastic anthropometric tape midway between the lower rib margin and the iliac crest [22]. Mid-upper-arm circumference of the right arm was measured at the midpoint distance between the bare acromion and olecranon, with the arm relaxed at the participant’s side [22]. All measurements were done in duplicate. Body mass index (BMI) was derived from weight and height (kg/m^2^), and thereafter BMI z-scores calculated based on growth reference algorithms developed by the World Health Organisation (WHO) for children and youth [25]. Definitions used to categorise children into underweight, healthy weight, overweight, and obese are shown in Table 1.

**Table 1:** Descriptions of cut-points used for outcomes of interest


**Physical activity**[26]	
Sedentary	< 100 CPM
Light	100 to 2999 CPM
Moderate	3000 to 5200 CPM
Vigorous	> 5200 CPM
**WHO BMI-for-age**[25]	
Underweight	< −2 SD
Healthy/normal	0.99 SD to −1.99 SD
Overweight	> +1 SD
Obese	> +2 SD

**Acronyms:** CPM (counts per minute); WHO (World Health Organization); SD (standard deviation); BMI (body mass index).

**Note:** The z-score system expresses the anthropometric value as a number of standard deviations or z-scores below or above the reference mean or median value.

### Direct measures (accelerometry)

Objective monitoring of physical activity, sedentary time, and sleep duration was achieved by use of ActiGraph GT3X + accelerometers at a one second epoch setting. Accelerometers were firmly attached to belts and distributed to the children who were then instructed to wear the instruments on the right side of their waists at all times except when bathing or swimming (including when sleeping). The research team demonstrated appropriate wearing of the accelerometers to ensure proper use. Participants were required to wear the devices for at least 7 consecutive days, plus an initial familiarisation day, in order to maximise the number of children providing a minimum of 4 days of wear of up to 10 hours, with at least one valid weekend day [22]. Non-wear time within a day was considered 60 consecutive minutes of ‘0’ counts. Reminder telephone calls and reminders from class teachers were helpful in ensuring compliance with the protocol and return of the accelerometers. Once accelerometers were returned, data were downloaded from the device to a computer, and the log reviewed for completeness [22]. Accelerometry data reduction was completed using cut-points developed by Treuth *et al*., which are validated in children and youth [26], and the relationships found confirmed using an alternate set of cut-points (Evenson et al. – data not reported) [27]. Time spent in sedentary, light, moderate, or vigorous physical activity was categorised as shown in Table 1.

### Questionnaires

A *diet and lifestyle questionnaire* related to dietary intake, physical activity, sedentary behaviours, and sleep duration was completed by participating children in the presence of research staff during school visits. A *demographic and family health questionnaire* captured information about the health history of the child, parental education, parent weight and height, and household income and facilities. A *neighbourhood questionnaire* was used to capture information on parental perceptions of the social environment, the neighbourhood built, food, and physical activity environment. A *tracing questionnaire* was used to collect detailed contact information for ease of follow-up. The three latter questionnaires were completed by the parents of participating children and sent back to the research team. A *school environment questionnaire* captured information on school characteristics, policies, and practices that may influence healthy eating and activity behaviours of the children. This questionnaire was completed by a school administrator or teacher in each of the participating schools. Lastly, one *school audit questionnaire* was completed for each participating school by the research study team. This questionnaire recorded directly observed information on the school’s built and food environments [22]. Full details of the questionnaires are proved elsewhere [22].

### Recruitment rates

A total of 1278 introduction letters and consent forms were distributed to children to take home to their parents or guardians. Of these, 634 (49.6%) participants were consented, and 563 (44.1%) participants completed all primary anthropometric assessments and questionnaires. Only 555 (43.4%) participants also had adequate accelerometry wear time. Participating children were 9.0 to 11.9 years of age, and were recruited from 16 public and 13 private schools throughout Nairobi.

### Statistical analysis

Descriptive statistics included means and standard deviation or frequencies. Univariable analysis was used to assess the associations between overweight/obesity or meeting the physical activity guidelines, with variables of interest selected *a priori* based on previous research findings and the socioecological model by Sallis *et al*. [14-20,22]. Statistically significant associations from univariable analyses were then used in multivariable analyses (logistic regression) to investigate the odds of being overweight/obese or meeting the physical activity guidelines in multi-predictor models. Hosmer-Lemeshow and c-statistic tests were used to ascertain that these multivariable models had good calibration and acceptable to excellent discrimination. All statistical analyses were computed using SAS version 9.3 (SAS Institute, Cary, North Carolina, USA).

## Results

### Participant and parent characteristics

Characteristics of participating children and their parents are summarised in Table 2. Of the 563 participants, 46.5% were boys and 53.5% were girls. Waist and mid-upper arm circumference data were presented in three tertiles with ranges and means for each in order to offer additional body composition descriptions of the sample. Based on WHO’s BMI z-score categorisation, we found that 3.7% of children were underweight, 75.5% were healthy weight, 14.4% were overweight, and 6.4% were obese (20.8% overweight/obese). The proportion of overweight/obesity was lower - 15.8% and 14.9% - when categorised based on Centers for Disease Control and Prevention (CDC) and International Obesity Task Force (IOTF) cut-points respectively [28,29]. Two hundred and forty five (45.7%) participants reported that they used active transportation (e.g. walking) to or from school. The mean daily, weekday, and weekend time spent in various activity intensity levels are also reported in Table 2. Notably, mean daily sedentary time was 398 minutes; mean daily time spent in light physical activity was 463 minutes; mean daily time spent in moderate physical activity was 32 minutes; and mean daily time spent in vigorous physical activity was a mere 4 minutes. Only 12.6% of participating children met the recommendation of ≥ 60 minutes of daily MVPA. The mean sleep duration was found to be 516 minutes (8.6 hours) for this sample of children.

**Table 2:** Descriptive characteristics of participating children

	**Mean (SD) or *n *** **(%)**
***Individual Factors***	
**Sex**	
Boys	262 (46.5%)
Girls	301 (53.5%)
**Waist Circumference (cm)**	
1st tertile (range: 47.85 – 58.30)	55.5 (1.9)
2nd tertile (range: 58.35 – 62.95)	60.5 (1.4)
3rd tertile (range: 63.00 – 108.10)	70.8 (7.9)
**Mid Upper Arm Circumference (cm)**	
1st tertile (range: 14.65 – 18.80)	17.4 (0.9)
2nd tertile (range: 18.85 – 21.20)	19.9 (0.7)
3rd tertile (range: 21.25 – 36.15)	24.5 (3.1)
**BMI Categories (WHO Cut-Points)**	
Underweight	21 (3.7%)
Healthy weight	425 (75.5%)
Overweight	81 (14.4%)
Obese	36 (6.4%)
**Transport to School**	
Active (Walking, bicycle, roller blade, skate board)	245 (45.7%)
Motorised (Bus, van, car)	291 (54.3%)
**Mean (minutes) in Various Activity Intensities**	
Daily minutes of Sedentary	397.8 (71.5)
Weekday minutes of Sedentary	419.5 (82.7)
Weekend day minutes of Sedentary	349.4 (94.9)
Daily minutes of Light	463.1 (69.8)
Daily minutes of Moderate	31.5 (20.2)
Daily minutes of Vigorous	4.0 (4.3)
Daily minutes of MVPA	35.4 (23.2)
Weekday minutes of MVPA	36.1 (24.2)
Weekend day minutes of MVPA	34.2 (28.7)
Met MVPA Guidelines *(Mean of ≥ 60 minutes of daily MVPA)*	71 (12.6%)
Mean (minutes) of Sleep Duration	516.4 (55.0)
***Parental Factors***	
**Maternal Education Level**	
Primary or less	91 (16.3%)
High school or less	163 (29.2%)
Diploma/Higher Diploma/Degree	237 (42.5%)
Graduate/Professional Degree	67 (12.0%)
**Paternal Education Level**	
Primary or less	43 (8.4%)
High school or less	159 (31.1%)
Diploma/Higher Diploma/Degree	197 (38.6%)
Graduate/Professional Degree	112 (21.9%)
**Maternal BMI (kg/m**^**2**^**)**	
Underweight (<18.50)	157 (27.9%)
Healthy weight (18.50 – 24.99)	128 (22.7%)
Overweight (25.00 – 29.99)	135 (24.0%)
Obese (≥30.0)	143 (25.4%)
**Paternal BMI (kg/m**^**2**^**)**	
Underweight (<18.50)	206 (36.6%)
Healthy weight (18.50 – 24.99)	142 (25.2%)
Overweight (25.00 – 29.99)	149 (26.5%)
Obese (≥30.0)	66 (11.7%)
***Household Environment Factors***	
**Total Annual Household Income (Kshs)**	
Low SES (<466,728)	264 (48.6%)
Mid SES (466,728 – 1,799,999)	177 (32.6%)
High SES (>1,800,000)	102 (18.8%)
***Neighbourhood Environment Factors***	
Trust (P*eople in my neighbourhood can be trusted*) -% that somewhat agree/strongly agree	301 (54.5%)
Getting Around (*There are shops/stores/markets and place to buy things that I need within easy walking distance*) -% that somewhat agree/strongly agree	467 (85.2%)
High crime rate -% that somewhat agree/strongly agree	232 (42.5%)
***School Environment Factors***	
**Participation by Type of School**	
Public	295 (52.4%)
Private	268 (47.6%)
Participants in schools with written policies (*underdevelopment or existing*) or practices on Physical Activity	529 (94.0%)
Participants in schools with written policies (*underdevelopment or existing*) or practices on Healthy Eating	475 (84.4%)

**Acronyms:** WHO (World Health Organisation); MVPA (moderate-to-vigorous physical activity); BMI (body mass index); SES (socioeconomic status); Kshs (Kenya shillings).

A higher proportion of fathers (21.9%) than mothers (12.0%) reported having attained a graduate or professional degree. More mothers (49.4%) than fathers (38.2%) were found to be overweight/obese based on WHO BMI categories as derived from self-reported height and weight variables [30]. Over half of parents (54.5%) agreed that they believed people in their neighbourhoods can be trusted. A large percentage (85.2%) reported that there were shops, stores, markets and places to buy the things that they required within easy walking distance. Less than half (42.5%) reported that there was a high crime rate in their neighbourhoods.

From the Government of Kenya tax brackets and through a consultative process, total annual household income was categorised into low, medium, and high SES. As expected, a majority of households (48.6%) fell in the low SES category, while 32.6% and 18.8% were medium and high SES respectively. Of the 563 participants, 52.4% attended public schools, while 47.6% were enrolled in private schools. A large proportion of children - 94.0% and 84.4% - attended schools with written policies or practices on physical activity and healthy eating respectively.

### Factors associated with overweight/obesity

As shown in Table 3, univariable analysis revealed that active transport, maternal education level, paternal education level, maternal BMI, paternal BMI, total annual household income, and type of school were all associated with being overweight/obese. A higher proportion of children using motorised transport (25.8%) were overweight/obese compared to those using active transport (14.7%) to get to/from school. As presented in Table 3, there was a progressive increase in the number of children who were overweight/obese with increasing maternal and paternal education attainment. Proportions of childhood overweight/obesity also increased with increasing household SES. Similarly, a higher percentage of children in private schools (33.6%) were overweight/obese compared to those in public schools (9.2%). Paternal BMI and type of school remained significant predictors of overweight/obesity in multivariable analyses as shown in Table 4. A one unit increase in paternal BMI was associated with a 17.2% increased odds of the child being overweight/obese. Attending a private rather than public school was associated with a 4.2 times higher odds of being overweight/obese.

**Table 3:** Univariable model for factors associated with childhood overweight/obesity and physical activity

	**Overweight/Obese (%)**	**p-value**	**Met daily MVPA guidelines (%)**	**p-value**
***Individual Factors***				
Sex				
Boys	54 (20.6%)	NS	46 (17.6%)	0.0013
Girls	63 (20.9%)		25 (8.3%)	
Transport to School				
Active	36 (14.7%)	0.0019	55 (22.4%)	< 0.0001
Motorised	75 (25.8%)		16 (5.5%)	
BMI Categories				
Overweight/Obese			3 (2.6%)	< 0.0001
Underweight/Healthy weight			68 (15.3%)	
***Parental Factors***				
Maternal Education Level				
Primary or less	3 (3.3%)	< 0.0001	34 (37.4%)	< 0.0001
High school or less	24 (14.7%)		23 (14.1%)	
Diploma/Higher diploma/Degree	69 (29.1%)		12 (5.1%)	
Graduate/Profession degree	21 (31.3%)		2 (3.0%)	
Paternal Education Level				
Primary or less	1 (2.3%)	< 0.0001	13 (30.2%)	< 0.0001
High school or less	14 (8.8%)		31 (19.5%)	
Diploma/Higher diploma/Degree	54 (27.4%)		9 (4.6%)	
Graduate/Profession degree	41 (36.6%)		7 (6.3%)	
Maternal BMI (kg/m^2^); *as continuous variable*	0.11 (r-value)	0.0297		NS
Paternal BMI (kg/m^2^); *as continuous variable*	0.27 (r-value)	< 0.0001		NS
***Household Environment Factors***				
Total Annual Household Income (Kshs)				
Low SES (<466,728)	27 (10.2%)	< 0.0001	59 (22.4%)	< 0.0001
Mid SES (466,728 - 1,799,999)	47 (26.6%)		10 (5.7%)	
High SES (>1,800,000)	35 (34.3%)		2 (2.0%)	
***School Environment Factors***				
Participation by Type of School				
Public	27 (9.2%)	< 0.0001	69 (23.4%)	< 0.0001
Private	90 (33.6%)		2 (0.8%)	

**Acronyms:** NS (not significant); BMI (body mass index); SES (socioeconomic status); Kshs (Kenya shillings).

**Note:** Spearman correlation coefficients presented for continuous variables.

**Table 4:** Multivariable model for factors associated with childhood overweight/obesity and physical activity

	**OR (Confidence limits)**	**p-value**
	**Overweight/Obese**	
Paternal BMI (kg/m^2^); *as continuous variable*	1.17 (1.09 – 1.26)	< 0.0001
Participation by Type of School *(Private vs. Public)*	4.18 (2.26 – 7.72)	< 0.0001
	**Met Daily MVPA Guidelines**	
Sex (Boys vs. *Girls*)	2.63 (1.49 – 4.64)	0.0009
Maternal Education Level		
*High school or less vs. Primary or less*	0.35 (0.19 – 0.67)	0.0014
*Diploma/Higher Diploma/Degree vs. Primary or less*	0.25 (0.12 – 0.54)	0.0008
*Graduate/Professional Degree vs. Primary or less*		NS
Participation by Type of School *(Private vs. Public)*	0.04 (0.01 – 0.17)	< 0.0001

**Acronyms:** BMI (body mass index).

**Note:** All variables that were significant in the univariable analyses (from Table 3) were included in the initial multivariable model, and a backward elimination approach used to reduce the model to only those that remained significant as reported in this table.

### Factors associated with meeting daily MVPA guidelines

As shown in Table 3, univariable analysis revealed that sex, active transport, child BMI category, maternal education level, paternal education level, total annual household income, and type of school were all associated with meeting physical activity guidelines. A higher percentage of boys (17.6%) than girls (8.3%) met the recommended daily minutes of MVPA. More children that used active transport (22.4%) met the physical activity guidelines compared to those that used motorised transport (5.5%). A higher proportion of underweight and healthy weight children (15.3%) met the guidelines compared to those in the overweight/obese category (2.6%). As presented in Table 3, there was a decreasing trend in the number of children who met the guidelines with increasing maternal and paternal education attainment. Proportions of children meeting the physical activity guidelines also decreased with increasing household SES. A considerably higher percentage of children in public schools (23.4%) met the guidelines compared to those in private schools (0.8%). Sex, maternal education level, and type of school remained significant predictors of meeting physical activity guidelines in multivariable analyses shown in Table 4. Boys were 2.6 times more likely to meet physical activity guidelines than girls. Mothers attaining a high school or lower education level (compared to a primary school or lower education level) was associated with a 64.8% decreased odds of their child meeting the guidelines, while mothers having a diploma, higher diploma, or degree (compared to a primary school or lower education level) was associated with a 75.1% decreased odds of their child meeting the physical activity guidelines. Children attending private schools were 96.4% less likely to meet physical activity guidelines than children attending public schools.

## Discussion

The objective of this study was to determine the prevalence of, and investigate factors associated with overweight/obesity and physical activity in school-aged children from Nairobi. The findings of this study were suggestive of the potentially negative impact of higher socioeconomic status and urbanisation on body weights and physical activity patterns of school aged children in this setting. These urban living children were found to have substantial proportions of overweight/obesity, high amounts of sedentary time, high volumes of light intensity physical activity, and considerably low amounts of time spent in MVPA.

### Individual level factors

Child under-nutrition – which includes underweight (low weight-for-age), stunting (low height-for-age), wasting (low weight-for-height), and deficiencies of essential vitamins and minerals [31] – remain some of Sub-Saharan Africa’s most fundamental challenges for improved human development [32-34]. This is a particular concern when considering the school-aged child population since malnutrition is associated with lower schooling attainment and educational performance, as well as shorter adult height and reduced adult income or economic productivity [31,32]. It has been suggested that when conducting studies on the prevalence of overweight/obesity in low and middle income countries, that stunting also be considered, since BMI works poorly when there is a high percentage of stunting [35,36]. The relationship between stunting and obesity is highly debatable, with some studies showing no relationship between the two [37,38], while others finding an existing relationship between the two [39,40]. For instance, even among child populations with high levels of stunting, stunting did not alter the relationship between BMI and other measures of body composition [38]. BMI was found to be the strongest predictor of all adiposity indicators, suggesting that BMI can be used in stunted populations in the same way as it is used in non-stunted populations [38]. While stunting refers to retarded linear growth as a result of chronic under-nutrition, wasting results from inadequate nutrition over a shorter period, and underweight encompasses both stunting and wasting [41]. Further, the high correlation between stunting and underweight suggests that the prevalence of underweight aptly describes the magnitude of the problem of growth faltering in children [41]. Consequently, it is probable that underweight was a reasonable indicator of child linear growth, particularly in this urban population where underweight was found to be low. Indeed, the criteria used to assess the nutritional status of children in SSA are important in estimating the prevalence of a nutritional double burden, defined as the coexistence of under-and-over nutrition in the same population [42]. This was indeed evident from the finding that even in the urban city of Nairobi, 3.7% of children were underweight. While under-nutrition continues to be an issue of critical importance in this region, the purpose of the present study was to examine the emergence of the other end of the spectrum – overweight/obesity – and the possible emergence of a nutritional double burden of public health challenges, as this may begin to place more strain on the limited healthcare resources in Kenya.

Data analysis revealed substantial proportions of childhood overweight/obesity for this age group. While these figures are lower than values in developed countries such as in the USA and Canada, which have childhood overweight/obesity prevalences of 33% and 29% respectively [43-45], these findings are indicative of an existing threat of childhood overweight/obesity among Kenya’s urban school-aged children. A recent systematic review examining the evidence for an overweight/obesity transition among school-aged children and youth in Sub-Saharan Africa found that weighted averages of overweight/obesity and obesity for the entire time period captured in the review were 10.6% and 2.5% respectively [46]. In light of the findings of this systematic review, these proportions of childhood overweight/obesity in Nairobi are indeed concerning. It is, however, noteworthy that the age range of the sample of children included in this study was 9 to 11 years, whereas those of the references cited were between 5 and 19 years. As such, there may be age differences in the prevalence of overweight/obesity not accounted for in these simplified comparisons.

Objectively measured mean daily sedentary time was 6.6 hours, with children spending more time in sedentary behaviours while at school than on weekends. This difference may be explained by the amount of time they were required to be seated in classrooms during the school week. Children also spent a considerable amount of time in light physical activity (7.7 hours). Objectively measured physical activity data in South African children 7 to 15 years of age similarly showed that they participated in a high volume of physical activity at low intensity [47]. Future studies should focus on investigating the potential health consequences of relatively large volumes of light intensity physical activity in this age group. The mean daily time spent in MVPA was 36 minutes, with only 12.6% of participating children meeting the recommendation of ≥ 60 minutes of daily MVPA. These findings are comparable to those of Ojiambo *et al*., who found that the mean daily time spent in MVPA by urban children from Eldoret town in Kenya (of somewhat lower socioeconomic status than Nairobi) was 44 minutes, with 16.5% of children meeting physical activity guidelines [18]. The proportion of urban children meeting the physical activity guidelines in Kenya is not much higher than some Western countries such as Canada, where objective measures revealed that only 7% of children and youth accumulated the recommended ≥ 60 minutes of daily MVPA [48]. A relatively high number of Kenyan children reported using active transport means to and from school. Active transport was associated with a lower likelihood of being overweight/obese, and a higher likelihood of meeting the physical activity guidelines. Unfortunately, numbers of children using active transport may be declining due to increased automobile availability and use, as well as declining road safety, which make active transportation difficult and dangerous for school children in many areas of Nairobi. Despite the cost effectiveness of active transportation, an increasing number of parents prefer that their children use school buses or personal cars for transport. It is important to note that active transportation may be an important strategy in maintaining or increasing physical activity levels in children from lower income countries, and this issue requires the attention of key stakeholders in order to inform future planning and policy development [49].

A significantly higher percentage of boys than girls met the recommended daily minutes of MVPA, and boys were more likely to meet physical activity guidelines than girls. Higher activity in boys has also been observed in the Canadian setting [48]. It was suggested that differences in gender roles (e.g. boys participating in higher energy expending roles or activities), and a higher motivation inherent in boys to participate in physical activity, may explain this sex difference [46].

### Parental factors

Higher paternal BMI was associated with increased odds of children being overweight/obese, and increasing maternal education level was associated with decreased odds of children meeting the physical activity guidelines. A study by Micklesfield *et al*. in South African children 11 to 15 years of age also showed that higher maternal socioeconomic status and education were associated with decreased childhood physical activity and increased sedentary time [50]. The results of this study therefore serve to provide further evidence of these relationships. However, it is important to note that there are various limitations associated with self-report of body height and weight by adults [51].

### Household and socioeconomic factors

Total annual household income and type of school (private vs. public) were markers of SES. Proportions of childhood overweight/obesity increased with increasing household SES and attending a private school (higher SES) rather than a public school (lower SES). Proportions of children meeting the physical activity guidelines decreased with increasing household SES and attending a private school rather than a public school.

Taken together, the findings of this study showed that higher SES was associated with higher body composition measures, pointing to a positive SES relationship with overweight/obesity. Higher SES was also associated with decreased time spent in MVPA, pointing to a negative SES relationship with physical activity. Higher parental education achievement, greater use of motorised transport, and attending private schools - all of which are more prevalent among and accessible to those in higher SES - were associated with a higher likelihood of children being overweight/obese and a lower likelihood of children meeting the physical activity recommendations. These findings are supportive of studies conducted in Kenya and other Sub-Saharan African countries showing similar deleterious associations between urbanisation and healthy active lifestyles and body weights in school-aged children [14-20,46,52,53]. Interestingly, they are the inverse of the relationship observed in many high income countries where studies have consistently shown that lower SES is associated with lower levels of physical activity, more time spent in sedentary behaviours, and higher risk for unhealthier lifestyles compared to higher SES children [54,55].

This research study had several limitations including a cross-sectional design, not being nationally representative, and the realisation that accelerometry does not directly measure habitual physical activity or sedentary behaviour; rather, estimates these categories based on movement counts and arbitrary intensity thresholds [56]. Nevertheless, this study provides robust objective measures of several healthy active living variables and related correlates among a large group of Kenyan school children and represents the first study of this kind in Kenya.

## Conclusions

The findings of this study were suggestive of the potential negative impact of higher socioeconomic status and urbanisation on body composition and physical activity patterns in school aged children in this setting. In order to tackle the growing concern of childhood overweight/obesity and inadequate physical activity in Kenya, we must first improve surveillance of these health risk factors. Herein lies the importance of conducting comprehensive research studies such as the ISCOLE study at a nationally representative level. While not nationally representative, it is envisaged that this ISCOLE-Kenya study may serve as a model for a nation-wide survey. Further, and strategies to address these growing threats should focus on higher SES and urban populations, with greater attention placed on girls, to ensure that children are attaining healthy active lifestyles.

## Abbreviations

MVPA: Moderate-to-vigorous physical activity; BMI: Body mass index; CDC: Centers for Disease Control and Prevention; IOTF: International Obesity Task Force; ISCOLE: International Study of Childhood Obesity, Lifestyle and Environment; SES: Socioeconomic status; WHO: World Health Organisation.

## Competing interests

The authors declare that they have no competing interests. ISCOLE is funded by the Coca-Cola Company. The funder had no role in study design, data collection and analysis, decision to publish, or preparation of the manuscript.

## Authors’ contributions

SKM, LMW, VOO, and MST contributed to preparations prior to implementing the study protocol in Kenya. SKM and LMW participated in data collection. SKM and MST analysed the data and interpreted the results. SKM led the writing of the manuscript. All authors contributed to the writing, editing, and approved this manuscript.

## Pre-publication history

The pre-publication history for this paper can be accessed here:

http://www.biomedcentral.com/1471-2458/14/436/prepub
